# Commentary: Alignment in social interactions

**DOI:** 10.3389/fpsyg.2017.01249

**Published:** 2017-07-26

**Authors:** Tom Froese, Leonardo Zapata-Fonseca

**Affiliations:** ^1^Departamento de Ciencias de la Computación, Instituto de Investigaciones en Matemáticas Aplicadas y en Sistemas, Universidad Nacional Autónoma de México Mexico City, Mexico; ^2^Centro de Ciencias de la Complejidad, Universidad Nacional Autónoma de México Mexico City, Mexico; ^3^Plan de Estudios Combinados en Medicina (MD/PhD), Facultad de Medicina, Universidad Nacional Autónoma de México Mexico City, Mexico

**Keywords:** social cognition, social interaction, interpersonal coordination, embodied cognition, enactive cognition

We welcome Gallotti et al. 's (2017) proposal to shift the study of social cognition from focusing on types of *representation* to types of *interaction*. This aligns with the enactive approach to social cognition (e.g., Froese and Di Paolo, 2011), which has long been arguing for this kind of shift (e.g., Varela, 2000; De Jaegher and Di Paolo, 2007). We offer some clarifications from this latter perspective, which will hopefully benefit the development of their proposal.

Gallotti et al. point to “reciprocal exchange of information” (p. 255) as the key criterion of social interaction. An immediate issue is that the term “information” is ambiguous. They mostly use it in a technical sense (e.g., following Friston and Frith's (2015) account of generalized synchrony), but there are semantic connotations (e.g., “systems reciprocate thoughts and experiences”, p. 255), an equivocation that is problematic (Hutto and Myin, 2013). Here we stick to the technical sense.

An example of reciprocity leading to mutual alignment are members of an audience clapping: hearing each other influences their clapping, thereby facilitating moments of spontaneous synchrony. In general, entrainment of coupled systems can indeed be explained by reciprocal information exchange, like two grandfather clocks synchronizing their pendulums via sonic influence (Oliveira and Melo, 2015). Thus, Gallotti et al. have rediscovered an important insight from the cybernetics era: there is a key difference between coupling two non-linear systems in a unidirectional (feedforward) and bidirectional (feedback) circuit, with the latter interaction giving rise to novel properties at the level of the integrated system.

More recently, this insight has been applied to the study of social interaction by several research programs, including in neuroscience (Schilbach et al., 2013), evolutionary robotics (Froese et al., 2013), and especially in psychology (Riley et al., 2011; Fusaroli and Tylén, 2015). They confirm that social interactions can only be fully understood at the collective level.

Gallotti et al. acknowledge some of these traditions, but surprisingly they do not refer to the perceptual crossing paradigm (Auvray and Rohde, 2012), an experimental setup designed for studying real-time interaction (see Figure 1A for an explanation) that can give rise to their five types of alignment (see their Figures 1–5). Especially their on-line types of alignment have been studied systematically, as exemplified by Figure 1B based on data taken from (Froese et al., 2014).

**Figure 1:**
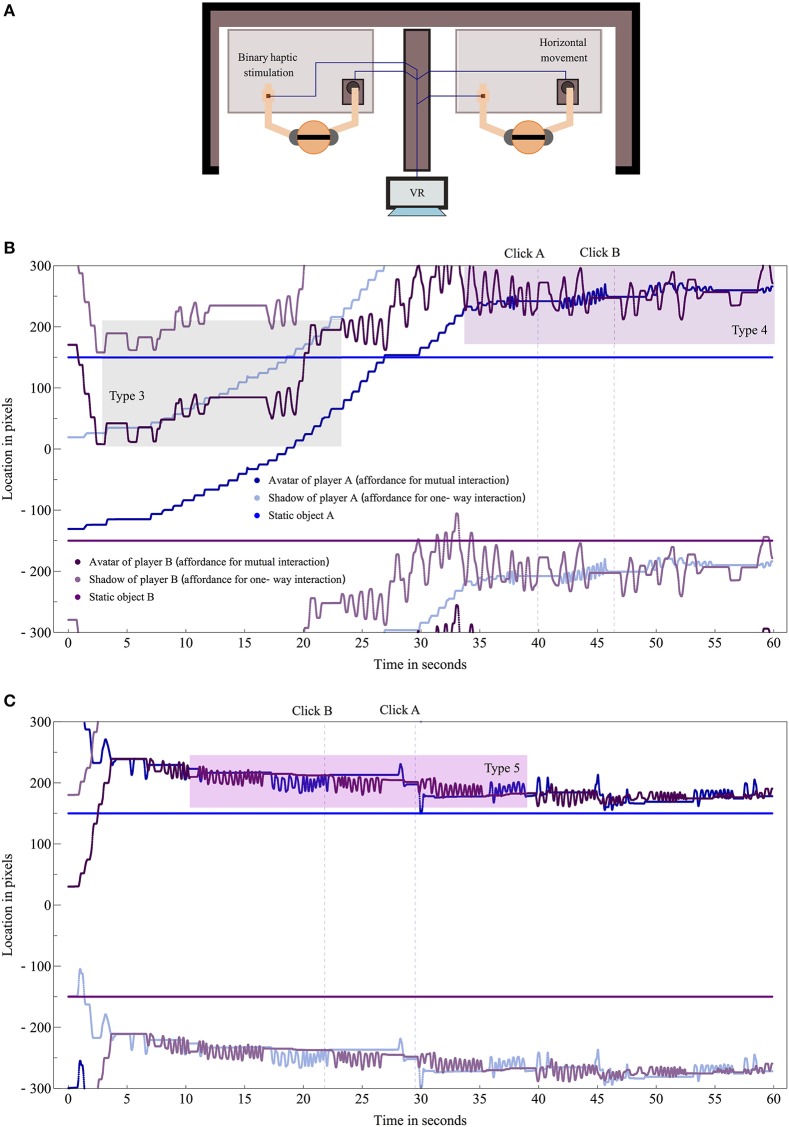
**(A)** Pairs of physically separated participants are embodied as avatars in an invisible virtual reality (VR) environment consisting of a line that wraps around. They can move their avatar and their hand will receive a tactile stimulation when it overlaps with another object. Each player can encounter a static object and two mobile objects: the other's avatar and a “shadow” that is copying the other's movements. The task for the players is to locate the other's avatar (and to mark this event by clicking). **(B)** Type 3: “on-line social cognition without mutual alignment” happens when A is moving and B is following A's shadow such that B's movements align with those of A but not vice versa (highlighted in gray). Type 4: “on-line social cognition with mutual alignment” happens when the avatars respond to each other's contact, e.g. by oscillating back and forth (highlighted in purple). Such perceptual crossing satisfies Gallotti et al.'s criterion for social interaction as reciprocal information exchange. A and B reported clear and almost clear awareness of the other, respectively, at the time of their click. **(C)** Type 5: type 4 situations in which it happens that one participant adapts more than another. Here A initially adapts more than B by imitating B's oscillatory movements (highlighted in pink). But the interaction then quickly gives rise to turn-taking and mutual imitation, which go beyond mere reciprocal dynamics because they depend on active co-regulation. Both participants reported clear awareness of the other at the time of their click.

In line with Gallotti et al.'s claim that the “pooling together of individual mental resources generates results that exceed the sum of the individual contributions” (p. 259), these experiments have repeatedly shown that players most frequently judge to be interacting socially precisely during mutual interaction (i.e., most clicks happen during type 4 and 5 situations). However, at the same time there is no statistically significant recognition of the other player, as measured in terms of individuals' sensitivity to social contingency for a particular virtual encounter (i.e., a player is equally likely to click following an encounter with the other's “avatar” and with the other's nonresponsive “shadow”). So instead this overall objective success has been explained at the collective level in terms of differences in the relative stability of the types of interactions (e.g., Di Paolo et al., 2008). This counterintuitive finding supports Gallotti et al.'s assertion that reciprocity can lead to mutual alignment, but is not sufficient for shared awareness (p. 256).

However, is this a case of online social cognition? Some have argued that it is (De Jaegher et al., 2010), but others have raised a number of concerns (e.g., Overgaard and Michael, 2015), which would similarly apply to Gallotti et al.'s proposal. Specifically, it is unclear whether an interactional factor that remains independent from an individual's judgments (or of their awareness of the other's presence) should count as cognitive (or social).

Froese and Di Paolo (2011) proposed the more conservative notion of a “multi-agent system” for such situations. They also argued that two additional criteria are required to go from dynamical reciprocity to social interaction: (1) they appeal to *normativity* to differentiate actions from physical happenings (thereby introducing a dependence on intentionality), and (2) they appeal to *co-regulation* to differentiate individually realizable actions from those requiring another responsive agent (thereby introducing a dependence on sociality). They also hypothesized that interactions satisfying these two criteria will give rise to social awareness.

The first criterion can be satisfied by reciprocal interactions in the perceptual crossing paradigm, but the second criterion may often remain unsatisfied, thus accounting for the lack of individual social sensitivity. Froese et al. (2014) investigated what happens when participants are encouraged to act collaboratively. They found that turn-taking emerged as a paradigmatic example of co-regulation, and that it was associated with clear social awareness shared by both participants (see Figure 1C). Other examples include mutual imitation and negotiating leader and follower roles.

Further analyses of the time series of participants' movements by Zapata-Fonseca et al. (2016) revealed they are characterized by complexity matching, which means that the clustering statistics of salient events followed a power-law distribution and that the scaling was similar across paired participants. They were of a similar form as has been found to support multi-scalar mutual alignment during vocal interactions (Abney et al., 2014). Yet complexity matching was not significantly associated with objectively more accurate social recognition or subjectively clearer social awareness. In other words, while mutual alignment can be understood as a relatively spontaneous property of a multi-agent sensorimotor loop, in which sensory information and motor activity are mutually adapting, it is not a sufficient marker of on-line social cognition *per se*. As suggested by Garrod and Pickering (2004), such lower-level automatic alignments are the substrate for higher-level dyadic properties to emerge.

In sum, reciprocal information exchange and mutual alignment are important aspects of bidirectional interactions, but Gallotti et al. require more conceptual resources to be able to explain how people “take one another into account” as other individuals. For the enactive approach the challenge is to account for the missing factors without falling back on appeals to internal mental representations and theory of mind (Hutto, 2008).

## Author contributions

TF wrote the initial draft of the manuscript, LZ added further comments and produced the figure. Both TF and LZ finalized the manuscript together.

### Conflict of interest statement

The authors declare that the research was conducted in the absence of any commercial or financial relationships that could be construed as a potential conflict of interest.
